# Size matters in telomere biology disorders ‒ expanding phenotypic spectrum in patients with long or short telomeres

**DOI:** 10.1186/s13053-023-00251-7

**Published:** 2023-05-15

**Authors:** Anna Byrjalsen, Anna Engell Brainin, Thomas Kromann Lund, Mette Klarskov Andersen, Anne Marie Jelsig

**Affiliations:** 1 Department of Clinical Genetics, Rigshospitalet, University Hospital of Copenhagen, Blegdamsvej 9, 2100 Copenhagen East, Denmark; 2 Department of Cardiology, Section for Lung Transplantation, Rigshospitalet, University Hospital of Copenhagen, Blegdamsvej 9, Copenhagen East, 2100 Denmark

**Keywords:** Telomere biology disorders, Lung fibrosis, Cancer predisposition, Long telomeres, Short telomeres

## Abstract

The end of each chromosome consists of a DNA region termed the telomeres. The telomeres serve as a protective shield against degradation of the coding DNA sequence, as the DNA strand inevitably ‒ with each cell division ‒ is shortened. Inherited genetic variants cause *telomere biology disorders* when located in genes (e.g. *DKC1*, *RTEL1*, *TERC*, *TERT*) playing a role in the function and maintenance of the telomeres. Subsequently patients with telomere biology disorders associated with both too short or too long telomeres have been recognized. Patients with telomere biology disorders associated with short telomeres are at increased risk of dyskeratosis congenita (nail dystrophy, oral leukoplakia, and hyper- or hypo-pigmentation of the skin), pulmonary fibrosis, hematologic disease (ranging from cytopenia to leukemia) and in rare cases very severe multiorgan manifestations and early death. Patients with telomere biology disorders associated with too long telomeres have in recent years been found to confer an increased risk of melanoma and chronic lymphocytic leukemia. Despite this, many patients have an apparently isolated manifestation rendering telomere biology disorders most likely underdiagnosed. The complexity of telomere biology disorders and many causative genes makes it difficult to design a surveillance program which will ensure identification of early onset disease manifestation without overtreatment.

## Introduction

Telomere Biology Disorders (TBD) comprise a group of inherited disorders characterized by a change in the function of the telomeres. Traditionally, TBD associated with short telomeres has been associated with the clinical entity *dyskeratosis congenita (DC).* This condition is characterized by a clinical triade of reticular pigmentation, oral leukoplakia, and nail dystrophy in combination with bone marrow failure (BMF) and/or pulmonal fibrosis [1, 2] often presenting in childhood or adolescence. Today, we know that the clinical phenotype is substantially broader and that TBD in some families present with isolated pulmonal fibrosis in adulthood with presumably no other affected family members. Additionally, our knowledge of genes associated with TBD is increasing, allowing for genetic testing of patients and their healthy relatives and enrollment in relevant surveillance. The frequency of TBD is unknown, but it is estimated that 50‒100 families in Denmark have TBD (population 6 M). This number is expected to rise as knowledge of TBD and yield of genetic testing increases. The purpose of this review is to present the biological background for TBDs, symptomatic hallmarks, and the use of genetic testing in the diagnostics of TBDs.

### Telomere function and lengths

**Fig. 1:**
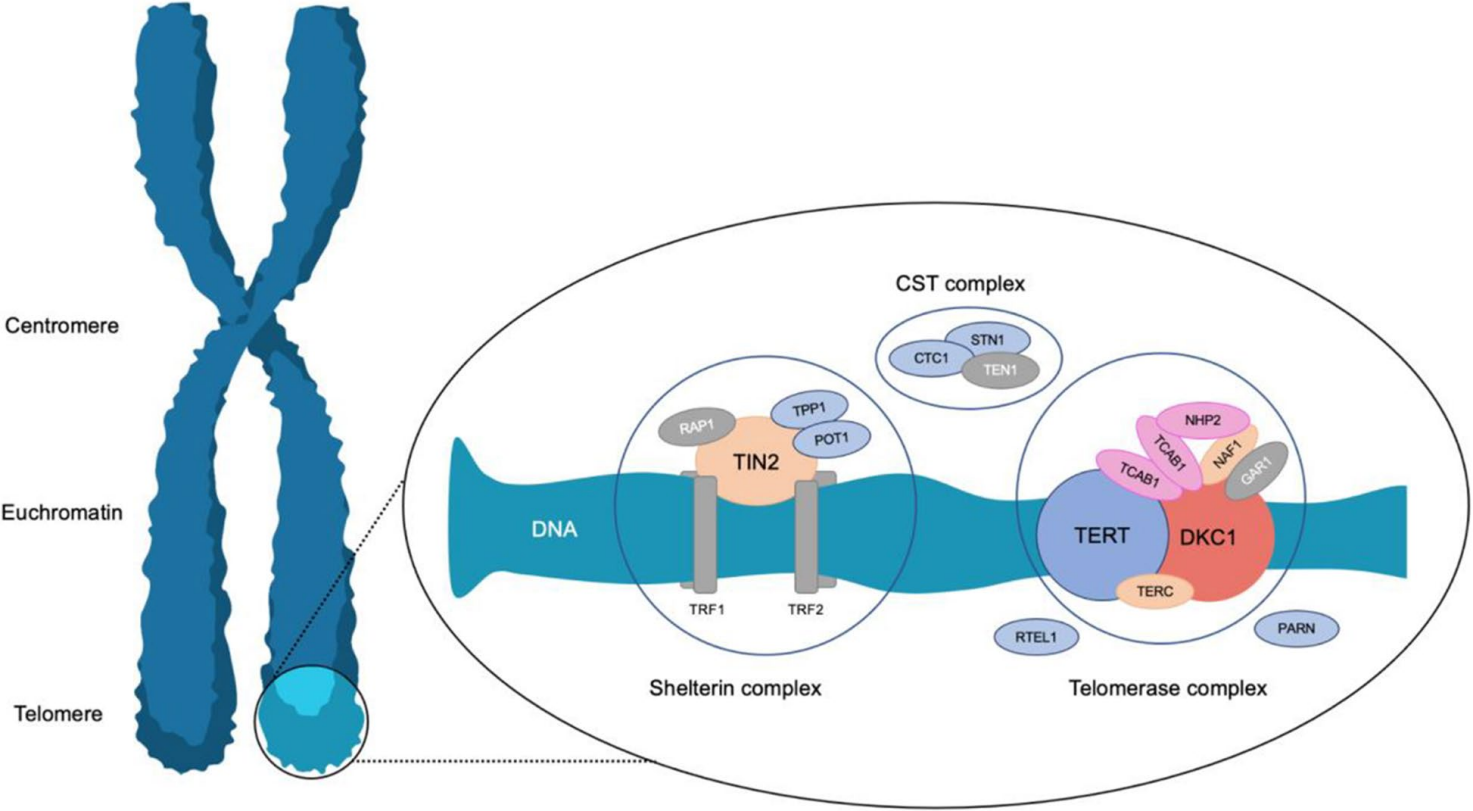
The components of the telomere. The outermost part of the telomeres is protected by a Shelterin complex consisting of six proteins: TRF1, TRF2, TIN2, POT1, TPP1 and RAP1. The telomeres are maintained at each cell division by a telomerase consisting of telomerase reverse transcriptase (TERT) and an RNA part that acts as a template when the nucleotides must be inserted during replication of the telomeres (TERC and DKC1). In addition, a number of proteins contribute to the maintenance and function of the telomeres. This applies to the CST complex (CTC1, STN1 and TEN1) and RTEL1, TCAB1 and PARN. The inheritance pattern is implicated by coloring. Pink: autosomal recessive inheritance; Yellow: autosomal dominant inheritance; Blue: autosomal recessive and autosomal dominant inheritance; Red: X-linked recessive inheritance. Proteins shown but not yet reported associated with TBD are colored in gray

Physiological function and length of the telomeres is crucial to maintain cell division throughout life. The telomeres are located at the ends of the chromosomes and consists of a double-stranded, non-coding, repetitive DNA sequence (TTAGGG)_n_ ending in a single stranded T-loop at the end of the chromosome. A protein complex (the Shelterin complex) ensures the stability of the telomere and protects the DNA strand against the regular repair mechanisms. The telomeres are protected by the DNA polymerase (telomerase) which protects the end of the telomeres against shortening and builds novel bases onto the telomere region (Fig. 1). Despite this effective system, the telomeres *will* be progressively shorter throughout life because of numerous cell divisions, with apoptosis and senescence of the cell as a result. There is biological variation of the length of the telomeres which is dependent on age, ethnicity, general hereditary, environmental factors, and type of cell [3].

TBD are caused by germline pathogenic variants in genes that encode the telomerase or the shelterin/protein complexes. These variants leads to critically short telomeres limiting the cells ability to replicate [2]. For most patients with TBD pathogenic variant(s) can be identified (Table 1) when performing genetic analysis from a blood sample, but in some instances no pathogenic variant can be identified, or the variant(s) identified are of uncertain significance. In the latter case, further genetic tests may be indicated to clarify the effect of the variant (if any).

**Table 1:** Telomere Biology Disorders (TBD) stratified by genetic etiology, syndrome, and clinical manifestation

	**Gene / protein or loci**	**OMIM no**	**Function related to telomere function**	**Inheritance**	**Clinical manifestation**
*Clinical telomere biology disorder and causative gene*					
Short telomeres	*CTC1*	#612199	3’ G-overhang regulation	AR	Dyskeratosis congenita^a^, Coats plus syndrome^b^
	*DKC1*	#305000	hTR stability	X-linked	Dyskeratosis congenita, Hoyeraal-Hreidarsson syndrome^c^, pulmonary fibrosis
	*NAF1*	*617868	hTR stability	AD	Pulmonary fibrosis, liver disease, myelodysplastic syndrome
	*NHP2*	#613987	hTR stability	AR	Dyskeratosis congenita
	*NOP10*	#224230	hTR stability	AR	Dyskeratosis congenita
	*PARN*	#616,353	hTR maturation and stability	AD, AR	Pulmonary fibrosis (AD), dyskeratosis congenita (AR), Hoyeraal-Hreidarsson syndrome (AR)
	*RTEL1*	#615190	Telomere replication, 3’ G-overhang regulation	AD, AR	Pulmonary fibrosis (AD), aplastic leukemia (AD), liver disease (AD), dyskeratosis congenita (AD), Hoyeraal-Hreidarsson syndrome (AR)
	*STN1*	#617341	3’ G-overhang regulation	AR	Coats plus syndrome
	*TERC*	#127550	Telomerase RNA scaffold, template	AD	Dyskeratosis congenita, aplastic anemia, pulmonary fibrosis, liver disease, myelodysplastic syndrome, acute myeloid leukemia, Hoyeraal-Hreidarsson syndrome
	*WRAP53* / TCAB1	#613988	Telomerase function	AR	Dyskeratosis congenita, Hoyeraal-Hreidarsson syndrome
	*ZCCHC8*	#618674	hTR stability	AD	Pulmonary fibrosis
Short and long telomeres	*ACD* / TPP1	#616553	Telomerase function	AD, AR	Short telomeres: Aplastic leukemia (AD), Hoyeraal-Hreidarsson syndrome (AR) Long telomeres: linked to melanoma and chronic lymphocytic leukemia
	*TERT*	#614742	Catalytic telomerase subunit, telomere elongation	AD, AR	Short telomeres: Pulmonary fibrosis (AD), aplastic leukemia (AD), liver disease (AD), myelodysplastic syndrome (AD), acute myeloid leukemia (AD), dyskeratosis congenita (AD/AR), Hoyeraal-Hreidarsson syndrome (AR) Long telomeres: melanoma (AD)
	*TINF2*	#613990	Regulation of telomere length	AD	Short telomeres: Dyskeratosis congenita, Hoyeraal-Hreidarsson syndrome, pulmonary fibrosis, Reversz’ syndrome^d^ Long telomeres: melanoma
	*POT1*	*609377	Telomerase activity, 3’ G-overhang regulation	AD, AR	(Primarily a long telomere syndrome, but listed here due to examples of homozygosity and a Coats plus like phenotype) Short telomeres: Coats plus syndrome (AR) Long telomeres: melanoma (AD), chronic lymphocytic leukemia (AD)
Long telomeres	*TERF2IP / RAP1*	*605061	‒	AD	Long telomeres: melanoma, chronic lymphocytic leukemia
*Genes linked to underlying telomere biology disorder*					
	*GRHL2*	*608576	‒	AR	Pathogenic variants cause poikiloderma with neutropenia and have an overlapping phenotype with dyskeratosis congenita but not short telomeres
	*LIG4*	*601837	‒	AR	Pathogenic variants cause poikiloderma with neutropenia and have an overlapping phenotype with dyskeratosis congenita but not short telomeres
	*USB1*	*613276	‒	AR	Pathogenic variants cause poikiloderma with neutropenia and have an overlapping phenotype with dyskeratosis congenita but not short telomeres
*Loci linked to altered telomere length through GWAS studies*					
	ACYP2, ATM, DCAF4, DCLRE1B, MPHOSPH6, PARP1, RFWD3, SON, TERF1, TERF2, ZNF208/ZNF257/ZNF676				

*AD:*  Autosomal dominant, *AR:* Autosomal recessive, *hTR:*  Human telomerase RNA

# number refering to disease/manifestation in OMIM

* number refering to protein function in OMIM

^a^Oral leukoplakia, changes in cutaneous pigmentation, nail dystrophy, pulmonary fibrosis, liver fibrosis, bone marrow suppression, hematologic cancers and squamous cell carcinoma of the head and neck

^b^Retinal disease, which result in retinal detachment and loss of vision in combination with leukodystrophy, ataxia, convulsions, and intellectual dysfunction. Osteopenia, anemia, and bleeding from the gastrointestinal tract are also seen

^c^Intrauterine growth retardation, bone marrow suppression, cerebellar hypoplasia, ataxia, microcephaly, malabsorption, and intestinal inflammation

Telomere lengths are thought to be similar in different tissues in fetuses and newborn children, but in adults the length differs between tissues [4]. However, over the last 10‒15 years standardized methods (telomere flow-FISH and qPCR) have been developed to assess the length of telomeres in different cell types including tumor tissue from cancer patients [5–7]. Assessment of telomere length using these methods can today be used in a clinical setting, where the length is mostly measured in distinct leukocyte lineages. Thus, a patient – suspected of harboring a TBD – can have a telomere length measurement performed to support the diagnosis or to indicate whether an identified genetic variant is of significance.. Yet, global telomere shortening is not always observed in patients with TDS, and, in general, results from telomere measurements analysis should be carefully interpreted in relation to the clinical presentation of the patient [7].

### Clinical spectrum

**Fig. 2:**
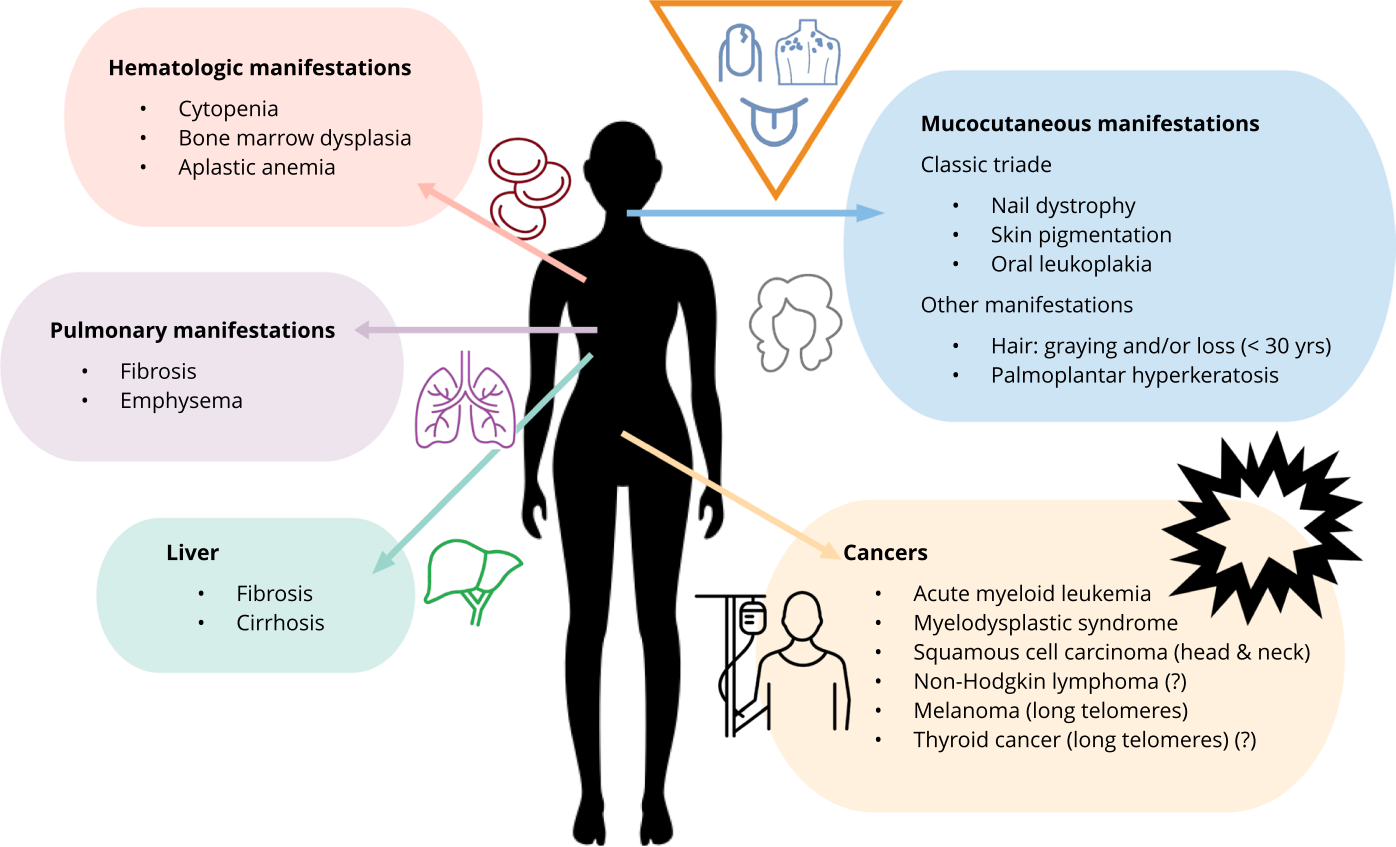
The most common clinical manifestations of telomere biology disorders (TBD). Arrows and icons show organ system affected by TBD

The clinical spectrum of TBDs is broad and the phenotype variable even within the same family. In the most severe cases, patients experience dysfunction of numerous organs within the first decade of life. The clinical entities *Hoyeraal-Hreidarsson syndrome*, *Revesz syndrome* and *Coats plus* are now known to be TBDs and represent the more severe end of the clinical spectrum. The syndromes are characterized by intrauterine growth retardation, cerebellar hypoplasia, cerebral calcification and bone marrow failure [1]. However, these syndromes are rare and traditionally TBDs have been associated with dermatological symptoms (oral leukoplakia, hyper- or hypopigmentation of the skin and nail dystrophy in association with early onset greying of hair) and BMF presenting in childhood or adolescence. Today, we know that this triade is only present in a subset of patients with TBD, and that the clinical spectrum is much broader, making TBD ‒ most likely ‒ underdiagnosed (Table 1 & Fig. 2). Isolated pulmonal fibrosis, typically presenting in adulthood, is found in some patients with TBD. Similarly, some adult patients present with bone marrow failure as the only manifestation. Furthermore, it is evident that some patients may experience very subtle or no symptoms and have a life span comparable to people without TBDs. Additionally, telomere length plays a role in immunologic function and aging mechanisms [8].

### Genetics

Sixteen genes have been associated with TBD and numerous genes/loci are linked to TBDs through genome wide association studies (GWAS) [9] (Table 1). The inheritance pattern can be either autosomal dominant, autosomal recessive or X-linked recessive. Family members risk inheriting a pathogenic germline variant from an affected parent, but the exact telomere length may differ within the same family. Pathogenic variants can be inherited from a parent, that perhaps is not affected, or occur sporadically (de novo) in the patient. This, in combination with the wide phenotypical spectrum, often results in a negative family history, despite identification of a pathogenic variant in the family. In some families an anticipatory inheritance pattern has been observed, in which age at first symptom occurs earlier and earlier from one generation to the next, and where the severity of disease also seems to worsen in the younger generations [10].

The wide phenotypical spectrum makes genetic counseling challenging. On one hand it is important that relevant family members are identified, but there is an inherent risk of overtreatment because of the variable penetrance. It seems reasonable to predictively test first degree relatives of affected patients and offer young relatives carrying the pathogenic variant surveillance. However, we lack long-term studies investigating the effects of surveillance. As of now this ‘erroring on the side of caution’ seems reasonable, but long-term studies of effects of surveillance are needed, and should in the future guide recommendations for surveillance.

### Hematologic manifestations

Bone marrow failure is a frequent manifestation in patients with TBD but is not pathognomonic (Fig. 2). Assessment of patients with classic DC shows that roughly 80% of patients have developed cytopenia at age 30 years [11, 12]. The bone marrow failure usually presents with cytopenia of one cell line which over time progresses to pancytopenia. The suspicion of a TBD should occur in the young cytopenic patient with hypoplastic bone marrow (including aplastic anemia and myelodysplastic syndrome, MDS), but may not phenotypically be distinguished from other inherited bone marrow failure syndromes. Cytopenia can progress to acute myeloid leukemia (AML). In a prospective cohort of DC patients Alter et al. found that at the age of 50 years about 50% of patients had developed bone marrow failure and 20% had developed MDS [13]. However, whether the frequency of hematologic manifestations is as high in patients without the classic DC-phenotype is unknown and might be a result of ascertainment bias in previous literature. Allogenic bone marrow transplantation is the only curative treatment of hematologic manifestations. Bone marrow transplantation is, however, not without complications in patients with TBD, as these patients are more sensitive to chemotherapy (and thus the preconditioning is given in a lower dose), their risk of cancer in other organs increases after transplantation and they more often develop graft vs. host disease compared to other patients undergoing bone marrow transplantation. Additionally, transplantation risks worsening pulmonal fibrosis in patients who present with both manifestations [1].

### Pulmonal manifestations

**Fig. 3:**
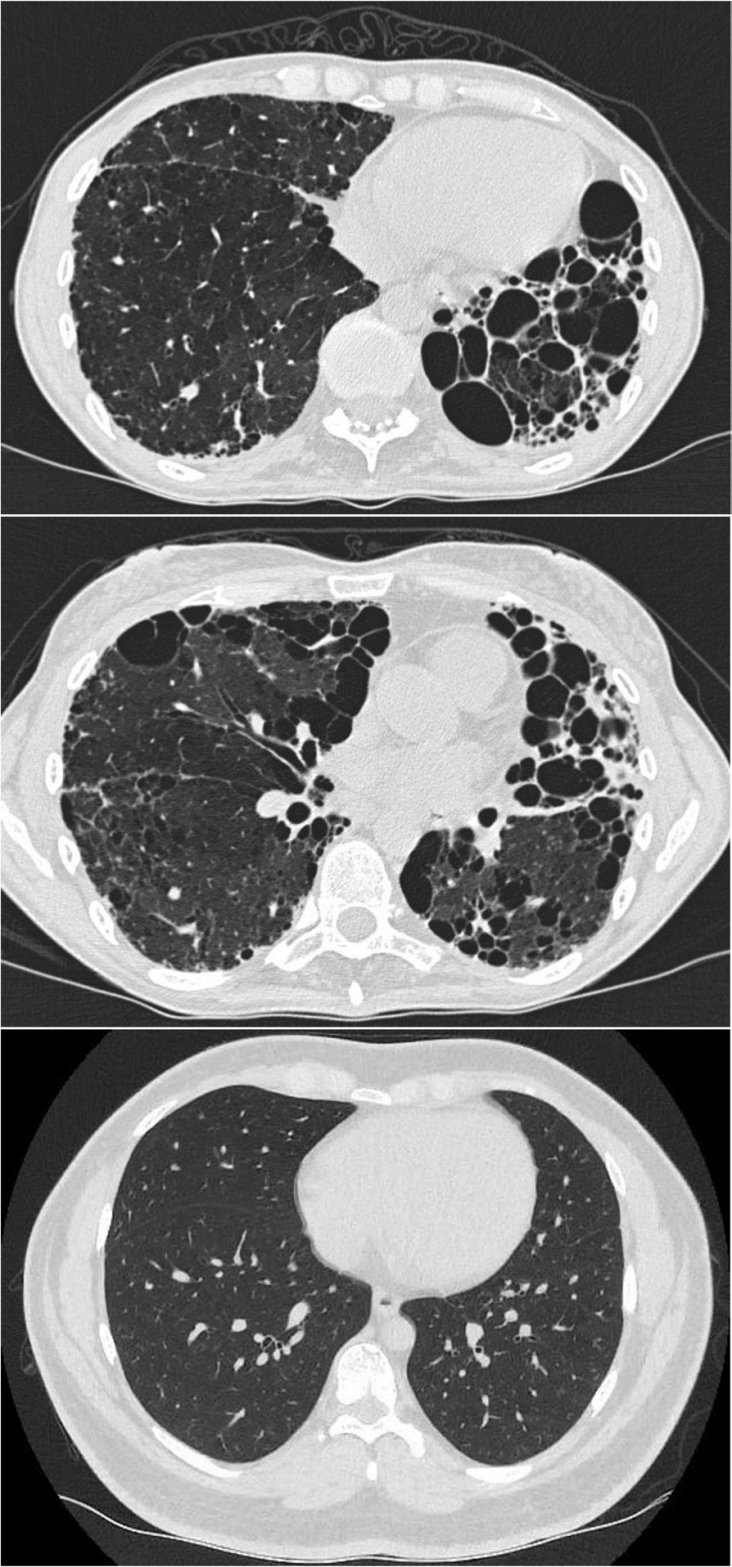
High Resolution Computerised Tomography (HRCT) showing lung fibrosis in a patient with a pathogenic variant in *TERC.* **A** and **B** Severe fibrotic and cystic changes in a patient with TBD and dyskeratosis congenita due to a pathogenic variant in *TERC* prior to lung transplantation. **C** HRCT in a patient with no lung disease and TBD

Previously it has been estimated that ~ 20% of patients with classic DC develop pulmonal fibrosis, however, some studies state that this is an underestimation of the actual number [11, 13, 14] (Fig. 3). In accordance a number of patients with isolated pulmonary fibrosis has in recent years been found to carry pathogenic variants in genes predisposing to TBD [15–17]. The patients are treated with antifibrotic treatment with Pirfenidon (a growth factor inhibitor) or Nintedanib (a tyrosine kinase inhibitor). The only curative treatment is lung transplantation. Yet, transplantation is often complicated by the risk of disease in other organs (BMF, liver cirrhosis), and patients more frequently present with primary graft dysfunction and chronic lung allograft dysfunction [18]. In addition, there is an increased risk of hematologic complications after lung transplantation [19]. Without treatment the survival from diagnosis is 2‒5 years [18, 20].

### Mucocutaneous manifestations

As previously mentioned, TBD can be accompanied by a triade of leukoplakia, poikiloderma (atrophic skin with reticular hypo- and hyperpigmentation and telangiectasia) and nail dystrophy.

Patients may present with early onset of grey hair (before age 30 years) as well as ophthalmologic symptoms (blepharitis, trichiasis and epiphora) [21].

### Cancer and other symptoms

In addition to an increased risk of hematologic malignancy patients with TBDs have a significantly increased risk of squamous cell carcinoma of the head and neck, often located in the tongue [13]. Head and neck cancers are primarily reported in patients with pathogenic germline variants in *DKC1* and seem to present before 50 years of age. The mechanisms for cancer development are unknown as is the risk of these cancers in patients, who do not carry germline *DKC1* variants. The risk for other types of cancer is unknown, but squamous cell anal cancer, non-Hodgkin lymphoma and basal cell carcinoma have been reported and the risk may be increased [11, 13]. TBD can also present with liver fibrosis/cirrhosis, portal hypertension and hepatopulmonary syndrome. Liver disease occurs in 5‒10% of patients with TBD [11, 22].

### Long telomeres – a clinical entity?

Pathogenic variants associated with short telomeres evidently have a health-related impact, however, although knowledge is limited, there is evidence that pathogenic variants associated with *long telomeres* also increase the risk of malignancy. However, the clinical presentation in patients with long telomeres is substantially different from that of patients with short telomeres. The hypothesis is that long telomeres provide a longevity advantage for the cell increasing the risk of malignancies [23]. In 2013, *Horn *et al. reported a family with several cases of malignant melanoma and a gain-of-function variant in *TERT* [24]. Subsequently, pathogenic variants in *TERT, TINF2, POT, ACD* and *RAP1/TERF2IP* have been reported in families with a high frequency of malignant melanoma, but without other clinical manifestations as seen in patients with short telomeres. Chronic lymphocytic leukemia (CLL), glioma and thyroid cancer has also been associated with long telomeres, but the association is somewhat unclear [25–29]. The penetrance in long TBDs is not complete and the precise risk of cancer is unknown.

### Suspicion of TBD and clinical work-up

TBD should be suspected in children/young adults with unexplained cytopenia, pulmonal fibrosis (before age 60), unexplained liver cirrhosis, mucocutaneus manifestations and/or early onset greying. A family pedigree showing other family members with similar symptoms will support such a suspicion. Work-up should include genetic testing of the 16 genes listed in Table 1. If a pathogenic variant is identified the family should be referred for genetic counseling, allowing for healthy relatives to be genetically tested and offered surveillance and options for family planning.

### Surveillance

Patients with TBD may be offered surveillance to identify early treatable manifestations and to avoid progression to high-risk disease [1, 30]. Suggestions of surveillance programs have been published but is difficult to design due to the variable phenotype and age of onset [1]. It does, however, seem reasonable that patients are offered hematologic, hepatologic and pulmonal surveillance, although there are no long-term follow-up studies confirming the effect of surveillance. Surveillance of the oral cavity, especially in patients with leukoplakia, may also be relevant to identify head-neck cancer [1, 31].

## Perspectives & conclusion

With the implementation of whole exome- and whole genome sequencing in clinical practice more variants in genes associated with TBD will invariably be identified, resulting in more patients being diagnosed with a TBD. Awareness of possible underlying TBD is important as early diagnosis will allow for identification of malignancy and pulmonary fibrosis at an early stage (and subsequent better treatment options and subsequent survival). The more recent finding that long telomeres confer an increased risk of some types of cancer is also important to design relevant surveillance for these patients. For both short and long TBDs it is important that studies investigate the effect of surveillance.

In conclusion, TBDs have proven to be more than a multiorgan syndromic disease manifesting both as isolated pulmonal fibrosis and in isolated hematologic disease, and awareness hereof is pivotal for correct diagnosis, surveillance and family planning in patients with TBD.

## Data Availability

Data sharing is not applicable to this article as no datasets were generated or analyzed during the current study.
